# The nociceptive withdrawal reflex does not adapt to joint position change and short-term motor practice

**DOI:** 10.12688/f1000research.2-158.v2

**Published:** 2013-12-19

**Authors:** Nathan Eckert, Zachary A Riley

**Affiliations:** 1 Department of Kinesiology & Program in Neural Science, Indiana University, Bloomington IN, 47405, USA; 2 Department of Kinesiology, Indiana University-Purdue University Indianapolis, Indianapolis IN, 46202, USA

## Abstract

The nociceptive withdrawal reflex is a protective mechanism to mediate interactions within a potentially dangerous environment. The reflex is formed by action-based sensory encoding during the early post-natal developmental period, and it is unknown if the protective motor function of the nociceptive withdrawal reflex in the human upper-limb is adaptable based on the configuration of the arm or if it can be modified by short-term practice of a similar or opposing motor action. In the present study, nociceptive withdrawal reflexes were evoked by a brief train of electrical stimuli applied to digit II, 1) in five different static arm positions and, 2) before and after motor practice that was opposite (EXT) or similar (FLEX) to the stereotyped withdrawal response, in 10 individuals. Withdrawal responses were quantified by the electromyography (EMG) reflex response in several upper limb muscles, and by the forces and moments recorded at the wrist. EMG onset latencies and response amplitudes were not significantly different across the arm positions or between the EXT and FLEX practice conditions, and the general direction of the withdrawal response was similar across arm positions. In addition, the force vectors were not different after practice in either the practice condition or between EXT and FLEX conditions. We conclude the withdrawal response is insensitive to changes in elbow or shoulder joint angles as well as remaining resistant to short-term adaptations from the practice of motor actions, resulting in a generalized limb withdrawal in each case. It is further hypothesized that the multisensory feedback is weighted differently in each arm position, but integrated to achieve a similar withdrawal response to safeguard against erroneous motor responses that could cause further harm. The results remain consistent with the concept that nociceptive withdrawal reflexes are shaped through long-term and not short-term action based sensory encoding.

## Introduction

Noxious electrical stimulation of the digits in humans produces a coordinated reflex response that results in the withdrawal of the limb, akin to removing the hand from touching a hot stove ^1, 2^. This reflexive action, known as the nociceptive withdrawal reflex ^3^, is an essential protective mechanism for interactions between parts of the body and the environment, allowing for quick removal from noxious stimuli. The relatively short-latency reflex responses occur as a result of transmission from Aδ afferent fibers to the motor neuron pools of several muscles ^4^. Previously, it was suggested that stimulation of these afferent fibers consistently produces excitatory postsynaptic potentials in flexor motor neurons and inhibitory postsynaptic potentials in extensor motor neurons of the cat hindlimb ^5^. However, fixed nociceptive input would limit the protective capabilities of the nervous system to only producing flexion at individual joints (e.g. elbow, shoulder), and a summary of more recent work has dispelled this hypothesis (see review by Clarke and Harris (2004) ^6^).

To initiate reflexive motor actions such as withdrawing the whole limb, nociceptive afferent input would need to be distributed to the motor neuron pools of muscles across several joints to coordinate the movement. In the hindlimb of rats activation of afferent neurons with receptive fields in a specific area of skin coordinates the activity of one or more muscles best suited to remove it from the noxious stimuli ^3, 7^. Specifically, the pairing of afferent neurons and resulting muscle activity was designated as reflex "modules" ^3, 7, 8^. Reflex modules are not limited to only activating the synergist muscles required to withdraw the limb, but can also inhibit muscles that would oppose it. Thus, organizing nociceptive sensory-motor interactions into reflex modules would result in a more efficient, coordinated limb withdrawal.

It has been suggested that the reflex modules are shaped by use, or action-based sensory encoding ^9, 10^. As a purely protective mechanism, the nociceptive withdrawal reflex adapts or develops according to the repetitive motor actions performed and the environment in which there are constant sensory-motor interactions. Since most of these adaptations transpire early in post-natal development ^11– 15^ it is assumed that they shape the functional sensory-motor behaviors required later in maturity ^14^. However, it is unknown how reflex modules are influenced by short-term action-based motor practice or use after development has ceased. Features such as habituation and dishabituation (wind-up), as well as human correlates of long-term potentiation (LTP) and long-term depression (LTD), have been well described in relation to short-term synaptic plasticity in nociceptive pathways ^2, 16– 19^. However, it is yet to be determined how short-term synaptic plasticity of nociceptive pathways controls the activation of reflex modules translating to the appropriate motor actions. Furthermore, the evidence presented for the activation of specific reflex modules has been demonstrated only by stimulating different receptive fields ^3, 7^.

The purpose of the present study is to test the adaptability of the nociceptive withdrawal reflex through two different tasks. First, the study seeks to determine if different nociceptive reflex modules are activated in the upper-limb of unimpaired humans by stimulating the same receptive field with the arm in different static arm positions by independently changing either the elbow or shoulder joint angles. We hypothesized that the static muscle length changes would alter group II afferent input that has been shown to have a strong excitatory input to interneurons regulating the withdrawal response ^20– 22^. Specifically, if there was modular organization in the spinal cord, we expected to observe different patterns of muscle activity (latencies, amplitude), and subsequent endpoint forces, evoked by stimulation in the various positions. Secondly, the study seeks to determine whether the protective motor function of the nociceptive withdrawal reflex in the upper-limb could be modified by short-term practice of a similar or opposing motor action voluntarily triggered by a non-noxious stimuli.

## Experimental procedure

### Participants

Ten healthy adults (7 males: 29.9 ± 7.5 yrs; range: 21–42 yrs) participated in the study. None of the subjects reported any neurological disorders or other upper limb musculoskeletal impairments. Each subject provided informed consent prior to participating. The protocol was approved by the Indiana University Institutional Review Board (Study # 1105005484) and was performed in accordance with the Declaration of Helsinki.

### Joint position task

**Figure 1.**
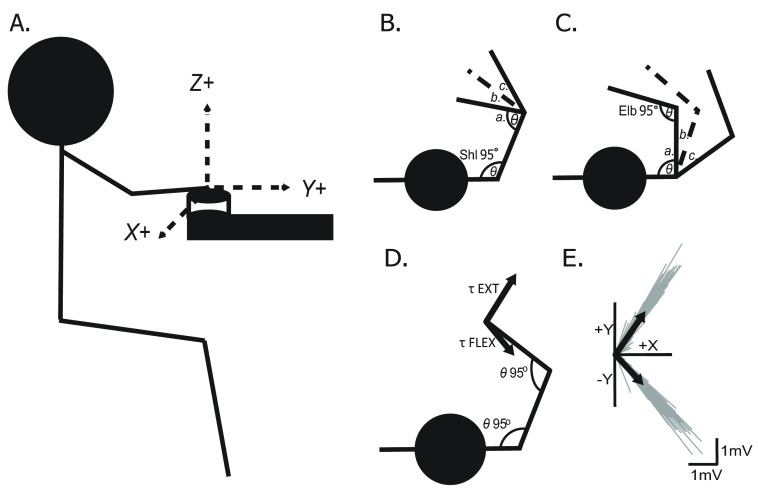
Schematic showing the joint configurations and details of the practice task. **A**) Sagittal view of the subject with the wrist secured to the force transducer and the orientation of the forces recorded for all of the experiments. **B**) When independently changing elbow angle (a. ELBFLEX; b. NEUT; c. ELBEXT) while keeping the same shoulder angle ( *θ* = 95°); and **C**) When independently changing shoulder angle (a. SHLFLEX; b. NEUT; c. SHLEXT) while keeping the same elbow angle ( *θ* = 95°). Note that the dashed line (b.) in both B and C is the same NEUT condition and was only tested once during the trials and also again after all of the joint positions had been completed. **D**) Schematic showing the joint configuration (τ = elbow ~95°, shoulder ~95°) for both EXT and FLEX practice protocols. **E**) Representative data from one subject demonstrating the magnitude and direction of the endpoint force vector from the nociceptive withdrawal response, after practice, from the individual trials (gray-thin lines) and the average responses (black-thick lines) in the (+/-)Y and +X directions. The average responses from the EXT and FLEX trials are also superimposed on panel D.

Nociceptive withdrawal reflexes were examined in five static conditions (see Figure 1A–C) where the arm was fully supported in front of the body in a general position similar to reaching to a stove top or opening a cabinet door. The five conditions were achieved by independently manipulating the elbow or shoulder joint angles in the transverse plane:

1) With the elbow flexed and the shoulder in a neutral position (ELB _FLEX_; *θ* = 75° and 95°, respectively);

2) Elbow extended and shoulder neutral (ELB _EXT_; *θ* = 115° and 95°, respectively);

3) Elbow and shoulder both neutral (NEUT; *θ* = 95° for both);

4) Shoulder flexed and elbow neutral (SHL _FLEX_; *θ* = 80° and 95°, respectively);

5) Shoulder extended and elbow neutral (SHL _EXT_; *θ* = 120° and 95°, respectively).

When the subject was fixed in each position shoulder abduction was ~75° and the only attachment point was at the wrist where it was affixed to the force transducer. This allowed the subject to completely relax the limb and did not require any stabilizing muscle activation from the shoulder or surrounding muscles. Each subject completed the five static conditions in a single experimental session, though the order of conditions was randomized for each subject. To examine if there was habituation in the withdrawal response the NEUT condition was repeated at the end of every experiment.

### Conditioning task

Nociceptive withdrawal reflexes were also examined in the right upper-limb of the subjects before and after two practice conditions performed on separate days. In this task the subject was in the aforementioned NEUT position for all of the trials. For the extension practice condition (EXT) the subject was asked to produce a submaximal, voluntary extension of their arm, as quickly as possible, against the force transducer upon receiving non-painful electrical stimulation of digit II (index finger). As the subject was secured at the wrist, this was an isometric contraction. This was repeated for 200 trials. The conditioning stimulations were delivered randomly with a period of 5–15s between stimuli in order to eliminate any fatiguing influence. The second condition, flexion practice (FLEX), was performed on a different day and required the subject to isometrically flex the arm at the elbow as quickly as possible. The experimental setup and endpoint force vectors generated by one subject in each practice condition are displayed in Figure 1D&E. Nociceptive withdrawal reflexes were assessed before and after the completion of the 200 trials in each condition. Shoulder and elbow flexion were kept at a constant ~95° and shoulder abduction was maintained at ~75° during each of the testing sessions. There was a minimum of two weeks between testing the two conditions and the order in which they were performed was randomized.

For all testing the subjects were comfortably seated in an upright position with only the right wrist secured to a six degree-of-freedom force transducer (75E20; JR-3, Woodland, CA), which kept them in a static position with each arm configuration. The JR-3 force transducer continuously sampled endpoint forces (Fx, Fy, Fz) and moments (Mx, My, Mz) during the experiments at 200 Hz. There were constant fluctuations in the gravitational force and moment (Fz, Mz) with the position of the limb in the two tasks, so this axis of force was excluded from further analysis.

### Stimulation conditions

For both the joint position task and the conditioning task noxious stimulation was applied using a Grass S88X stimulator (Grass Technologies, Astro-Med, USA) connected in-series with a Digitimer DS7AH constant current electrical stimulator (Digitimer LTD, UK). Short trains of electrical stimuli were delivered to digit II using stainless steel ring electrodes secured to the medial and proximal phalanges, while the subject was at rest. Each stimulus train consisted of 10 pulses (200 μs duration) delivered at 300 Hz, which was consistent with previous research evoking withdrawal reflexes in the upper limb at rest ^2^. Electrical current from the stimulator was slowly increased to determine perceptual threshold at the beginning of the experiment. Then it was increased in ~2 mA steps (relative to perceptual threshold) until the subject reported that further increases were intolerable. This resulted in a stimulus intensity of between 30–50 mA for all subjects, which was consistent with other studies in the upper limb ^1, 23, 24^. The stimulus trains used to elicit the reflex response during the experimental protocols were delivered at random intervals between 5 and 15s. Eight stimulus trains were delivered in all experiments where the nociceptive withdrawal reflex was examined. Stimulation intensity remained constant across all conditions for a given subject within each day of testing.

In the conditioning task non-noxious stimulation for the 200 practice trials was performed at an intensity of 5–15 mA (1.5 × perceptual threshold). The same stimulation trains were delivered during the practice trials, only at a much lower intensity that was not sufficient to evoke a noticeable short-latency reflex in any of the muscles. Stimulus trains were delivered randomly between 5 and 15s for the practice trials as well. Five minutes of rest was given between the initial nociceptive withdrawal reflex testing and the practice trials, and between the practice trials and nociceptive withdrawal post-testing.

### Electromyography

Surface electromyography (EMG) signals were recorded the same in both tasks, with single differential bar electrodes (Delsys Inc, MA, USA). The signals were amplified and conditioned using a 16-channel Bagnoli EMG System (Delsys) with high- and low-pass cut-off frequencies of 20 Hz and 1,000 Hz, respectively, before being stored at a final gain of 1,000 Hz with Spike2 software (CED, Cambridge, UK). Surface EMG activity was recorded from the abductor pollicus brevis (APB), brachioradialis (BRD), biceps brachii long head (BBL), triceps brachii lateral head (TRI), anterior deltoid (AD), and posterior deltoid (PD) muscles in the right upper limb. The right (ESR) and left erector spinae (ESL) were also recorded to monitor postural responses. The skin overlying each muscle was cleaned prior to affixing the electrode over the individual muscle belly. A single reference electrode was placed over the acromion process.

### Data analysis

Forces and moments were considered relative to subject coordinates with the +X direction pointing to the right of the subject, the –Y direction pointing towards the subject, and the +Z direction pointing upwards. Analysis of the endpoint forces and moments were confined to the horizontal (X-Y) plane. For all of the nociceptive withdrawal reflex trials the resultant two-dimensional (X-Y) endpoint force vectors were computed from the local peak force up to 200ms after stimulation. EMG for each muscle was processed by first removing the DC offset; then rectifying the signal. The latencies for withdrawal reflex onsets were designated when the reflex response exceeded a threshold of three standard deviations (SDs) above the mean EMG amplitude during a 100ms pre-stimulus baseline period (-100ms-stimulation). Withdrawal reflex offsets were determined based on previous observations of voluntary withdrawal latencies, such that the response reported was limited to the nociceptive spinal pathways ^25^. Withdrawal reflex responses in the upper-limb muscles were quantified by calculating the mean EMG for a time window between onset and offset latencies. The analyses were performed on each individual stimulation trial and the resulting values were averaged across the 8-stimuli for statistical comparisons. In addition, resultant two-dimensional (X-Y) endpoint force vectors were computed from the local peak force up to 350ms for the 200 FLEX and EXT practice trials.

### Statistical analysis

In the joint position task analysis of variance (ANOVA) was used to compare the direction and magnitude of the reflex endpoint force vectors across all arm positions. Arm position was considered as an independent factor and subject as a random factor for all multiple comparisons. Mean EMG reflex responses and onset latencies for each muscle were compared with separate ANOVAs across all positions. Bonferroni adjustments were made to correct for multiple comparisons in any additional post-hoc analysis. The forces, moments, EMG response amplitudes, and EMG onset latencies were compared between the initial and follow-up NEUT conditions, examining habituation, with independent t-tests.

The conditioning task was examined using paired t-tests to compare the direction and magnitude of the endpoint force vectors, peak forces and moments, and mean EMG reflex responses and onset latencies before and after each set of practice trials. To enable comparisons between the EXT and FLEX conditions that were tested on different days the data was expressed as a percent change from pre-post practice ( *i*_2_- *i*_1_/ *i*_1_*100) within each condition. The percent change in the direction and magnitude of the endpoint force vectors, peak forces and moments, and mean EMG reflex responses were then compared between EXT and FLEX conditions with independent t-tests. Onset latencies were compared between EXT and FLEX conditions as the absolute change from pre-post (ms). All data processing and statistical analyses were performed in MATLAB (Mathworks, MA, USA). Results were considered significant if *p* < 0.05.

## Results

### Joint position task

**Figure 2.**
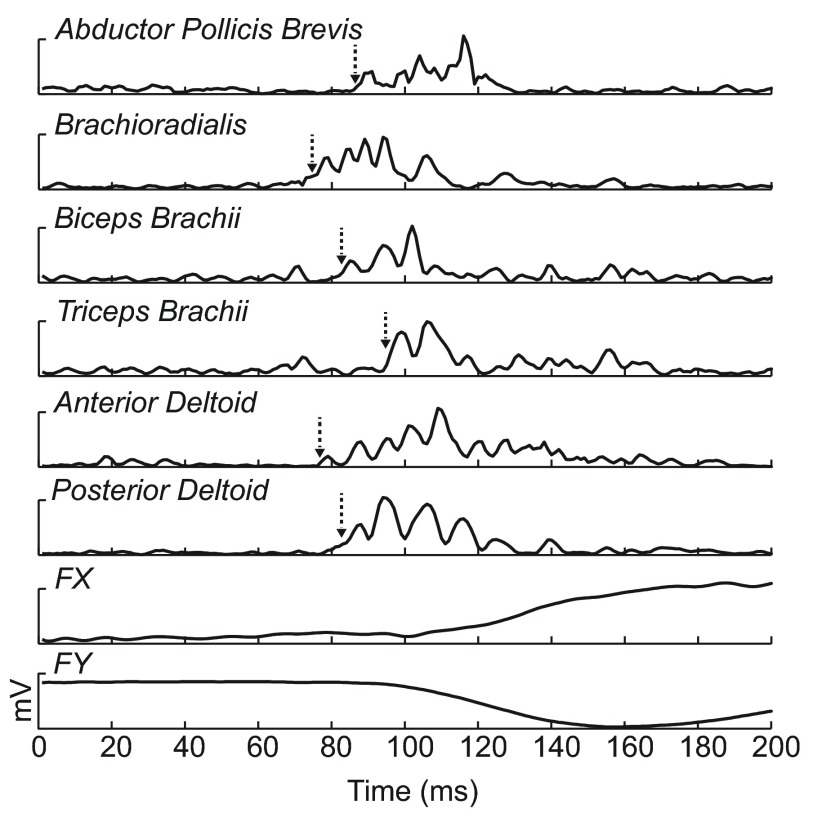
Representative data from one subject in the NEUT position where each trace represents the average of 8 individual stimulation responses. The average onset time is indicated by the dashed line with the arrow. The DC offset was removed and the signals were rectified prior to averaging for each channel. The time window is from the stimulation onset-200ms.

The average withdrawal response evoked in the NEUT position for one subject is presented in Figure 2. Specifically, the withdrawal responses from all muscles except the erector spinae are displayed, along with the corresponding X- and Y-endpoint force traces. There were never any clear responses in the erector spinae, so that data is not presented. There were no differences in any of the EMG or force variables between the initial and follow-up NEUT position testing, suggesting there was minimal habituation to the stimulus, or at the least that the stimulation and recording conditions did not change. Onset latencies for the withdrawal responses were not significantly different across the arm positions for any of the muscles recorded. In addition, the amplitude of the withdrawal EMG response was not different across positions for any of the muscles shown in the grouped data.

**Figure 3.**
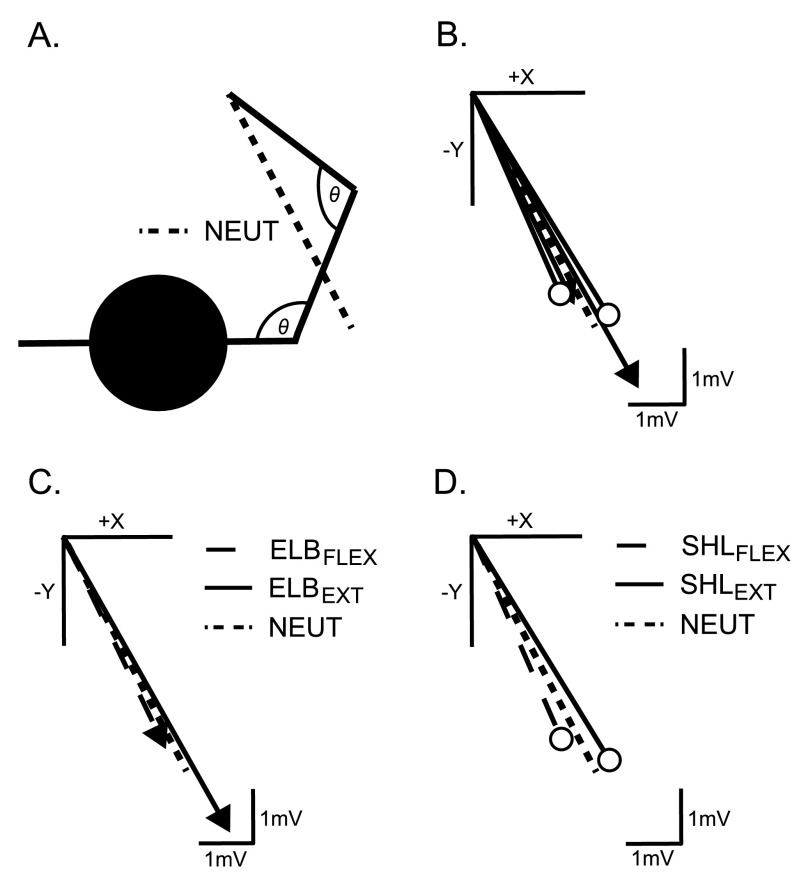
Results from the endpoint force vectors. **A**) Representative figure showing the arm in the NEUT position and the corresponding group force vector response to stimulation. **B**) Average force vector for all subjects in each position. The dashed line is the NEUT position. The lines with arrows are for the independent manipulation of the elbow angle, while the lines with circles correspond to changing shoulder angle. The same data from panel **B** is displayed in panels **C** and **D**, separated for the elbow and shoulder positions, respectively.

The individual endpoint forces (Fx, Fy) and moments (Mx, My) were not different across positions. When the endpoint force vectors were calculated from the peak Fx (+x) and Fy (-y) magnitudes (posterior-lateral direction, see Figure 3), significant differences were present in the direction of withdrawal between the SHL _FLEX_ and SHL _EXT_ positions ( *p* < 0.05). In addition, the magnitude of the endpoint force vector was significantly greater in the ELB _EXT_ position than in the ELB _FLEX_ or SHL _FLEX_ positions ( *p* = 0.025).

### Conditioning task

**Figure 4.**
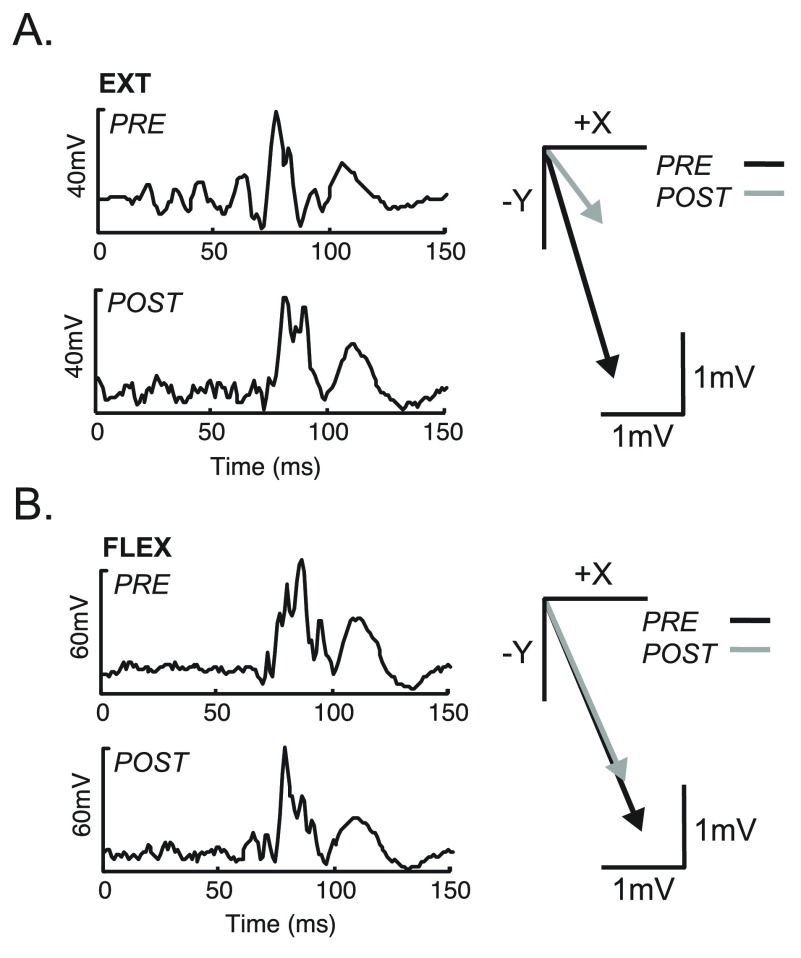
Representative nociceptive withdrawal responses before and after practice from the posterior deltoid (PD) muscle of one subject as well as the average direction and magnitude of the same subjects endpoint force vector for both A) EXT and B) FLEX practice.

The average withdrawal reflex response before and after EXT and FLEX practice is shown for the PD muscle and the endpoint force vectors for one subject in Figure 4. The only significant group difference observed in reflex onset latency after EXT practice was in the PD muscle where the response was observed at 78 ± 11ms before practice and 84 ± 18ms after EXT practice ( *p* = 0.001). In the FLEX condition the reflex onset latency was significantly delayed after practice in the BRD (86 ± 12ms–94 ± 18ms, *p* = 0.012), but no significant differences were noted for any other muscle in the FLEX condition. Furthermore, there were no significant differences in the pre-post absolute change in reflex latency (ms) between the EXT and FLEX conditions. Table 1 displays the variability in onset latency changes pre-post practice for each of the conditions. In addition, no differences were present in the EMG reflex response amplitudes for any of the muscles before and after practice, for either EXT or FLEX conditions. Similarly, there were no differences between the EXT and FLEX practice conditions, with the exception of the AD muscle that showed a significantly greater decrease in the EMG reflex response in the FLEX condition ( *p* = 0.037).

**Table 1.** Mean change (+ increase, - decrease) of onset latencies from the electromyography (EMG) response after EXT and FLEX practice for each muscle and all subjects. The muscles listed are the biceps brachii long head (BBL), triceps brachii (TRI), brachioradialis (BRD), posterior deltoid (PD), anterior deltoid (AD), abductor pollicis brevis (APB), right erector spinae (ESR), and left erector spinae (ESL).

	Subject 1		Subject 2		Subject 3		Subject 4		Subject 5		Subject 6		Subject 7		Subject 8		Subject 9		Subject 10	
	EXT	FLEX																		
BBL	+	+	+	+	−	+	−	+	−	+	−	+	−	−	+	+	+	−	+	−
TRI	+	−	+	+	+	+	−	+	−	−	+	−	−	−	−	−	+	+	+	−
BRD	−	+	+	+	+	+	+	+	−	−	+	−	−	−	−	+	+	−	−	+
PD	−	−	+	+	+	+	−	+	−	+	−	−	+	+	+	+	+	+	+	−
AD	+	+	−	+	+	+	+	−	−	+	−	−	−	+	+	+	−	+	−	+
APB	+	+	+	+	+	−	+	+	+	+	−	−	+	+	−	+	−	−	−	+
ESR	+	−	+	+	+	+	−	−	−	−	+	+	+	−	−	+	+	−	+	+
ESL	+	−	+	−	−	−	+	−	−	−	−	+	+	−	+	−	−	−	−	−

**Figure 5.**
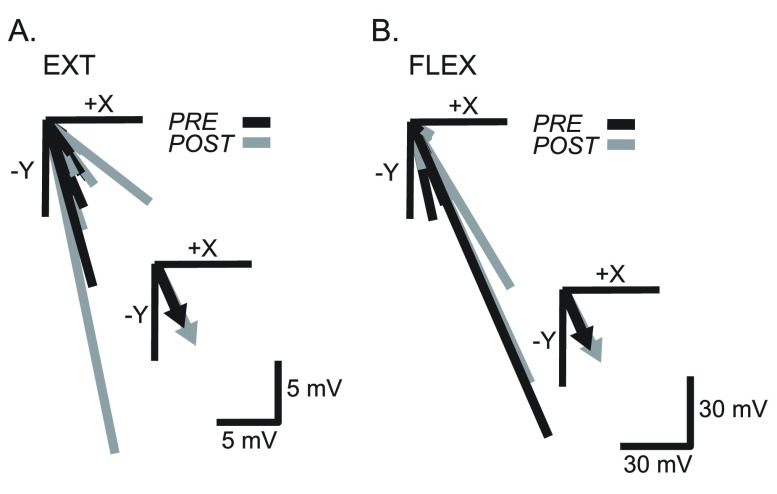
Group endpoint force vectors from the Fx (+x) and Fy (-y) before (black) and after (grey) practice for each subject with the A) EXT practice, and the B) FLEX practice. The inset on each panel shows the average for all subjects. No significant differences were noted.

The individual endpoint peak forces and moments were not significantly different before and after either EXT or FLEX practice with the exception of the My-moment (pitch), which was significantly lower after EXT practice ( *p* = 0.007). There were no differences in the direction or magnitude of the endpoint force vectors calculated from the peak Fx (+x) and Fy (-y) magnitudes (posterior-lateral direction) pre-post practice within either condition, or between EXT and FLEX conditions ( Figure 5).

Electromyography and force recordings of nociceptive withdrawal responses during both joint manipulation and conditioning tasks. Data associated with the joint manipulation task can be found in Exp1_position folder and is separated by the subject’s number and joint, elbow or (Sub#_Elb) shoulder (Sub#_Shl), and the static position of the joint (extended (ext)/flexed (flex)/neutral (neut)) during the evoked withdrawal response. Exp2_cond provides data associated with the resultant withdrawal response pre- and post- conditioning task and is separated by subject number and the isometric voluntary action of elbow extension (Sub#_Cond) or flexion (Sub#_Flex) in response to the 200 conditioning stimuli.

## Discussion

The purpose of the present study was to examine the adaptability of the nociceptive withdrawal reflex across different tasks. First, changing the elbow or shoulder joint angles independently resulted in a consistent nociceptive withdrawal response to the stimulation of the same receptive field on digit II. This conclusion is based on the pattern of withdrawal reflex activity in the upper-limb muscles being similar, regardless of the position of the limb. Some statistical differences were observed in the withdrawal force vectors, however, these differences did not correspond to changes in withdrawal reflex muscle activity across positions, suggesting they were due to altered limb biomechanics. More importantly, the direction of the withdrawal force response was relatively constant no matter the configuration of the upper-limb (see Figure 3). This was even the case when re-testing the NEUT position at the end of the experiment, where adjustments in limb position and repeated noxious stimuli did not show habituation in the nociceptive reflex, which has been observed previously ^2^. Second, the nociceptive withdrawal response was resistant to change, at least to the short-term motor practice performed. While there were a few significant differences observed in individual EMG and force variables, the main observation was that the reflex responses were not consistently modified by either EXT or FLEX practice. If the nociceptive withdrawal reflexes in the upper limb are considered merely a protective mechanism then it would be expected that the response would be robust. These results lead us to conclude that the action-based shaping of nociceptive reflex modules in the spinal cord help to prevent erroneous adaptations that would potentially compromise safety.

Much of the work examining the organization of nociceptive withdrawal reflexes has demonstrated distinct excitatory and inhibitory nociceptive reflex responses depending on the receptive fields stimulated (see review by Clarke and Harris (2004) ^6^). In the present study the withdrawal reflexes only elicited consistent excitatory responses in the upper-limb muscles regardless of the joint angle that was altered. This result is incongruent with the notion that the synergist muscles removing the limb should be excited while the opposing muscles should be inhibited ^3^. It is difficult to ascertain the benefit of co-activating muscles throughout the entire upper limb in response to noxious stimulation, particularly if it slows down the physical withdrawal of the limb. Co-activation of the muscles throughout the upper limb in response to nociceptive stimulation could be specific to the spinal motor neurons being relatively quiescent during these trials, as reported previously ^26^. There is also the potential that EMG cross-talk between opposing muscles (e.g. biceps brachii and triceps brachii) could have obscured smaller changes such as suppression, in particular with the muscle already quiescent ^27^. Alternatively, there are distinct excitatory and inhibitory responses in upper limb muscles during movement, even demonstrating dependence on the phase of movement ^26, 28^. It has been suggested that tactile afferent input is organized by the actions the system performs routinely; for example, reaching and withdrawing the limb voluntarily ^10^. If this nociceptive organization is linked to development ^13^, or is action-based, it can be reasoned that the nociceptive withdrawal response at rest would be much less finely-tuned or that the synaptic input would be largely dispersed in the spinal cord. Specific to the present study, this rationale would also explain the similar directions of withdrawal for each of the arm configurations.

The original hypothesis that the nociceptive withdrawal reflex response would vary with the joint position was based on the expectation that each joint position would have an optimal withdrawal motor response and that the static muscle length changes around the elbow or shoulder joints would alter group II afferent input to interneurons that help to gate the withdrawal response ^20, 21^. It appears this assumption was too generalized since it is known that group II afferents from secondary muscle spindle endings have a relatively constant discharge rate and low discharge rate variability in static postures ^29– 31^; and thus the difference in group II afferent feedback may not have been sufficient to modulate nociceptive input. It has been suggested that the nociceptive withdrawal response arises from the integration of multiple sensory feedback sources, and that there is a complex sensorimotor transformation mediating the appropriate motor response ^8, 10, 15^. In this case the group II afferent feedback alone would just be part of a larger, more complex set of sensory inputs that might be weighted differently across individual joints and arm positions. This would be feasible since there is a large amount of redundancy in multi-joint control of the upper limb and it has been reported that weighted sensory feedback in the upper limb improves control of the limb ^32^. The five positions examined in the present study only encompassed a small area of the workspace of the upper limb, even confined to a single plane, so in this context very little weighting of sensory feedback would even be required to produce the same withdrawal response.

Nociceptive input is widely distributed to motorneurons innervating muscles across several joints and with different primary movements ^8^, and because of this it was originally hypothesized that short-term motor practice would modulate the withdrawal reflex response. However, the results of the present study suggest that the motor practice the subjects performed was unable to alter this distribution of input. Presumably, this was because the practice was non-specific to the nociceptive withdrawal reflex, meaning that the subjects were practicing a voluntary motor task. However, the results of this study have caused us to re-examine the actual benefit of short-term plasticity in the nociceptive withdrawal reflex. Assuming the withdrawal reflex is organized with reflex modules, it is necessary for the reflex modules encoding nociceptive input to be developed over time and based on the motor actions commonly performed by the individual ^3, 7, 8, 10, 14, 15, 33, 34^. This being the case, short-term adaptations would likely compromise the built-in protective capabilities of the nociceptive system. This is not to suggest that habituation and dishabituation (wind-up) of the nociceptive pain pathways does not occur ^2, 16– 19^, but rather that the musculotopic organization underlying the activation of specific reflex modules ^9^, ultimately producing the motor action, is relatively insensitive to short-term practice. This remains in agreement with the demonstrated results, however the task utilized in the current study may fail to elicit plasticity within the withdrawal reflex.

The results presented suggest potential environmental factors that must be taken into account. The timeline for the purported action-based shaping of reflex modules remains unclear. As mentioned previously, the model of action-based sensory encoding or somatosensory imprinting has been described in early post-natal development ^13, 15^, where spinal organization can be determined by simple tactile feedback from spontaneous muscle twitches during sleep ^35, 36^. However, the mature nociceptive system is much less flexible, presumably because withdrawal receptive fields have already been optimized to eliminate or depress erroneous connections and strengthen appropriate connections ^11, 12^. Consequently, to override, or adapt the adult nociceptive reflex modules to new biomechanical, anatomical, or action-based constraints there would need to be a much greater amount of practice. It has been suggested that the long-term plasticity occurs as a result of a greater transfer of information between primary motor cortex (M1)-basal ganglia-cerebellum ^37^. Alternatively, the initial phase of adaptation can occur in as little as 10–30 minutes of practice, and can be driven by the disfacilitation of intracortical circuits in M1 ^38, 39^. Consequently, the short-term motor practice in the current study could have been specifically targeting the primary motor cortex and was not sufficient to cause adaptations in cerebellar circuits and pathways mediated through the cerebellum.

The original purpose of the present study was to examine the adaptability of the nociceptive withdrawal reflex across different tasks. The manipulation of joint position and reflex conditioning would activate specific reflex modules that would result in an efficient, coordinated limb withdrawal distinctly different from the control. The present results do not directly support the notion that different reflex modules were activated by independently changing elbow or shoulder joint angles. However, this does not suggest that there is no modular organization in the spinal cord for nociceptive input; rather it could just provide additional evidence that the reflex modules are specific to the receptive field activated. Instead, we hypothesize that the current data implies there is an additional layer of complexity to the sensorimotor transformation of nociception, meaning that the convergence of weighted multisensory input in the spinal cord was integrated to activate the same reflex module(s) for each arm position. In addition, since there appears to be a specific reflex encoder for each muscle in the dorsal horn of the spinal cord ^34^, the integration of multisensory input had to be similar for each muscle to result in the same pattern of activation. Chang *et al.* ^40^ recently demonstrated that whole limb kinematics are preferentially preserved over individual joint kinematics following peripheral nerve injury in the hindlimb of cats, and a similar compensatory mechanism may be responsible for the preservation of the withdrawal force direction despite altering individual joint angles. Thus, while there appears to be very complex processing of nociceptive input through interneuronal spinal circuits, the limb is consistently withdrawn in a general posterior direction towards the body. This could be a natural consequence of development ^15, 35^, or could be specific to non weight-bearing muscles that receive fewer cross-spinal inputs. Though it has been demonstrated repeatedly that the nociceptive withdrawal system is not hardwired, as was first believed ^5^, the results of the present study imply that the nociceptive reflex modules are also resistant to short-term adaptations from practicing motor actions. This is consistent with the protective function of the nociceptive withdrawal reflex, as it guards the body from erroneous responses that could cause further harm. The data collected in the current study demonstrates a level of complexity within the nociceptive withdrawal reflex that was previously unclear. The results suggest that the motor response of the nociceptive withdrawal reflex remains fixed, which is in agreement with the long-term development of nociceptive reflexes, and regardless of the specific mechanisms involved, the nociceptive withdrawal response in the upper limb serves as an efficient means of protecting the hand.
