# Supplementary material for: The nociceptive withdrawal reflex does not adapt to joint position change and short-term motor practice
Source: F1000Res. 2013 Dec 19;2:158. Originally published 2013 Jul 16. [Version 2] doi: 10.12688/f1000research.2-158.v2 (PMC3886789; doi:10.12688/f1000research.2-158.v2)
Supplement: Peer review history

# Peer review: Referee response for version 1

**Published:** 2013-12-10

**Authors:** Brach Poston

**Affiliations:** 1 Cleveland Clinic Lerner Research Institute, Cleveland, OH, USA

The purpose of the present study was to examine the adaptability of the nociceptive withdrawal reflex in different task conditions after motor practice. Specifically, this was accomplished by applying electrical stimulation to the second digit in a total of five different arm positions (shoulder and elbow joint angle combinations), both before and after motor practice performed either in the opposite or same direction as the stereotyped withdrawal response.

The primary dependent measures of interest were the force responses recorded at the wrist and the EMG reflex responses obtained from six muscles of the upper limb. The main findings were:

1. The amplitude and onset latencies of the EMG reflex responses were similar in all of the arm configurations for every muscle studied.
2. There were minimal differences in the direction of the withdrawal force response for the different arm configurations.  
3. A single session of motor practice in either the opposite or same direction as the stereotyped withdrawal response did not change the pattern of the EMG or Force responses.

The authors concluded that the nociceptive withdrawal response is resistant to short-term motor practice. Thus, if it is even possible to significantly modify the withdrawal reflex response, it would likely take extensive motor practice over a long period of time, which makes intuitive sense as it would not be beneficial for protective reflex responses to be modified by short-term perturbations. Therefore, it seems that the author’s interpretation of their findings is appropriate.

 

Overall, the manuscript is very well-written, the data appear to have been collected carefully, and there are no major flaws or concerns, although the authors could probably have shortened the Introduction and Discussion sections.

The topic addressed seems to be relatively novel as I don’t believe any previous studies in humans have addressed the degree to which the nociceptive withdrawal reflex can be modified in these task conditions with motor practice. Therefore, the current work makes a significant contribution to the current body of literature on the topic.

To the credit of the authors, they presented a balanced and justified interpretation of the results even though some of the findings did not match their original hypotheses. In addition, they pointed out any limitations of their study, which will have to be addressed in future research.

Finally, the focus of the research seems to be appropriate for F1000 Research and of interest to many readers of the journal.

I have read this submission. I believe that I have an appropriate level of expertise to confirm that it is of an acceptable scientific standard.

# Peer review: Referee response for version 1

**Published:** 2013-12-02

**Authors:** Hong-You Ge

**Affiliations:** 1 Center for Sensory-Motor Interaction, Aalborg University, Aalborg East, Denmark

The title of the article “ *Adaptability of the nociceptive withdrawal reflex*” is not so specific to the current study. The title of the article should be confined to the main research results of the study, i.e., the nociceptive withdrawal reflex responses in the upper limb are resistant to the change of joint position and short term motor practice.

The abstract represents a suitable summary of the work. The experimental design and methods are appropriate and the discussion is well balanced. The conclusion is justified on the basis of the results of the study.

I have read this submission. I believe that I have an appropriate level of expertise to confirm that it is of an acceptable scientific standard.
